# Design of high SERS sensitive substrates based on branched Ti nanorods

**DOI:** 10.1038/s41598-022-15875-3

**Published:** 2022-07-08

**Authors:** Nosirudeen Abayomi M. Yussuf, Jianlin Li, Yung Joon Jung, Hanchen Huang

**Affiliations:** 1 Department of Mechanical and Industrial Engineering, Northeastern University, Boston, MA 02115 USA; 2 Department of Mechanical Engineering, University of North Texas, Denton, TX 76203 USA

**Keywords:** Nanoscale materials, Nanoscience and technology, Nanoscale devices, Nanoscale materials

## Abstract

This paper reports a rational design of branched titanium (Ti) nanorods formed by glancing angle physical vapor deposition and their applications as substrates for surface-enhanced Raman scattering (SERS). Ti nanorods with branches have larger surface areas than non-branched nanorods. However, Ti surface oxidizes easily resulting in very little SERS effect. The SERS sensitivity of the branched titanium nanorod is improved by annealing Ti nanorods in nitrogen in an effort to reduce oxidation. Additionally, the plasmonic resonance of the branched titanium nanorod is further improved by coating the top of the nanorods and branches with silver (Ag). The sensitivity of the SERS substrates is about 3700% that of as-deposited branched Ti nanorods with a native oxide layer. Our investigation provides a mechanism to fabricate sensitive SERS sensors of Ti nanorods that are known to be thermally and chemically stable and compatible with silicon-based electronics.

## Introduction

Surface plasmon resonance involves the collective oscillation of free electrons around the nuclei in the subwavelength surface when light is incident on a metal nanostructure^1^. This oscillation in various modes mediates local field enhancement close to nanostructured noble metal surfaces, providing a surface enhancement effect, such as surface-enhanced Raman scattering (SERS)^2,3^. SERS is a non-destructive yet powerful tool for ultrasensitive vibrational spectroscopy, and It has been applied in the detection of chemical and biological agents on nanostructured surfaces since 1970^4,5^.

Branched Ti nanorods from glancing angle deposition (GLAD) are one-dimensional nanostructures that result in a large surface area to volume ratio^6^ compared to non-branched nanorods. Once exposed to air, the surface of Ti nanorods oxidizes to form TiO_2_. This oxide is thermally more stable than Ti and reduces coarsening of Ti nanorods, thereby reducing the degradation of SERS sensitivity in high-temperature environments^7–10^. However, when used as SERS substrates^11^, they exhibit SERS signals with relatively low sensitivity than noble metals. For SERS applications, it is desirable for these nanorods to not oxidize easily, possess high thermal stability and high SERS sensitivity similar to noble metals like Ag and Au^12–17^. Scientists in previous research^18^ have shown that TiN exhibits localized field enhancement similar to those obtained using nobel metals. In addition, TiN is thermally stable and can be produced using the method of Ti nitridation. Therefore, the branched Ti nanorod with a large surface area coated with TiN is proposed to enhance the SERS sensitivity of the substrate while the coating of a small amount of noble metal additionally, results in very high SERS sensitivity.

In this paper, we experimentally design branched titanium nanorods capped with Ag and investigate their sensing performance, with and without nitridation treatment. For our investigations, we choose methylene blue (MB) as the probe molecule. MB has been widely used by many research groups^19–25^ and is extensively applied in industries and household products since it has a high Raman signal and can be easily adsorbed on the metal surface. The results indicate that the branched Ti nanorod treated in a nitrogen atmosphere and coated with 50 nm of Ag exhibit the highest SERS enhancement.

## Results and discussion

**Figure 1:**
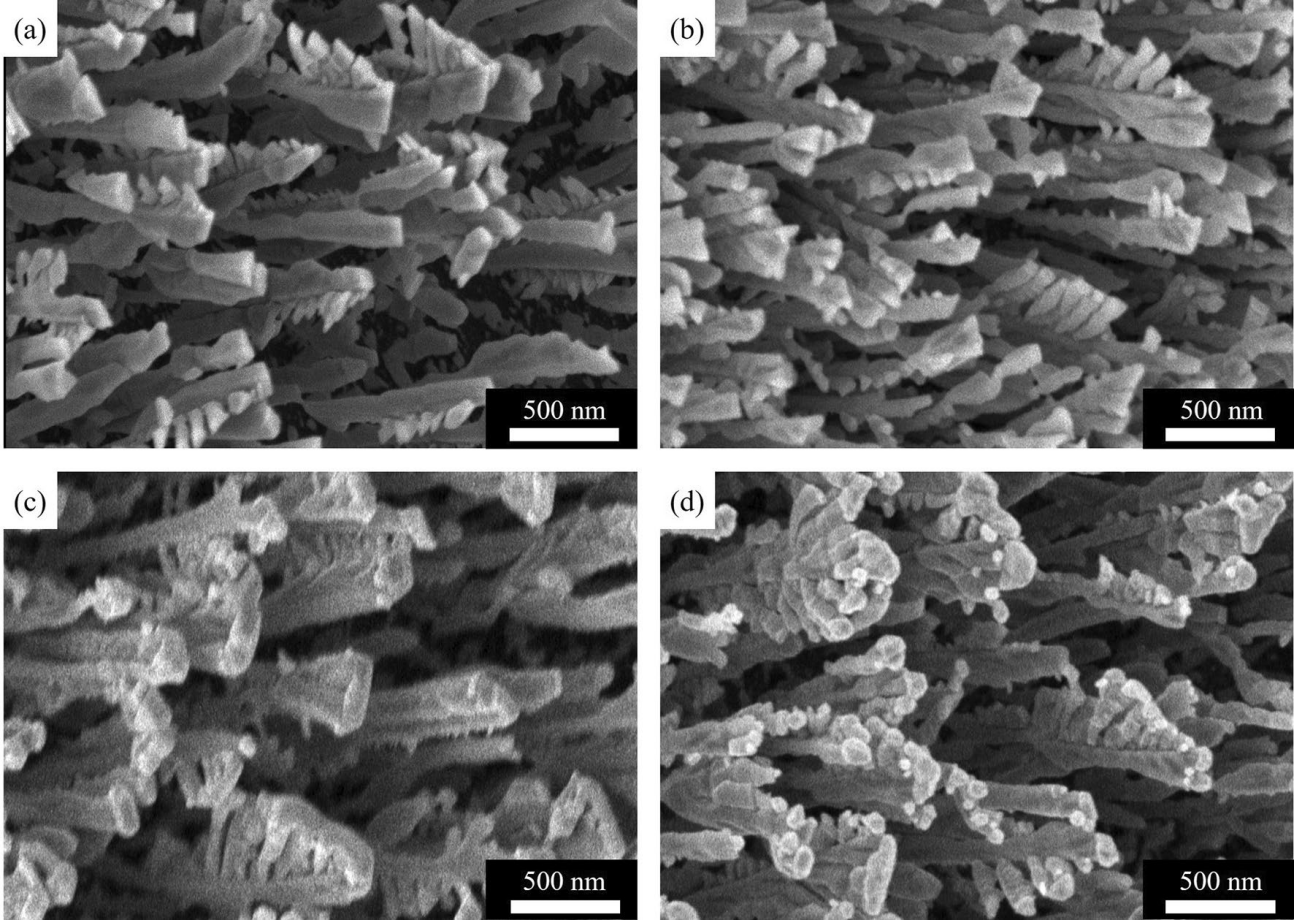
SEM images of Ti nanorods, (**a**) as-deposited Ti, (**b**) annealed for 4 h in an N_2_ environment, (**c**) capped with 100 nm of Ag on as-deposited Ti, (**d**) capped with 100 nm of Ag on Ti annealed for 4 h in N_2_.

As the first set of results, we present the scanning electron microscope (SEM) images of as-deposited Ti nanorods compared with Ti nanorods annealed in N_2_ gas (here on denoted as Ti–TiN), Ti nanorods capped with Ag (here on denoted as Ti–AgX, where X is the Ag cap nominal thickness) and Ti nanorods annealed in N_2_ gas and capped with Ag (here on denoted as Ti–TiN–AgX, where X is the Ag cap nominal thickness). As shown in Fig. 1, the morphology of the branched Ti nanorods does not present any apparent changes, especially within the Ti and Ti–TiN samples in Fig. 1a,b. The measured diameter of the nanorods is within 150–300 nm, which is comparable to previous work^6^. Figure 1c,d shows that the deposition of 100 nm Ag leads to total coverage of the top of the nanorods and bridging of the top of the nanorod branches, and in some cases, bridging at the top of the nanorods and the top of the branches.

**Figure 2:**
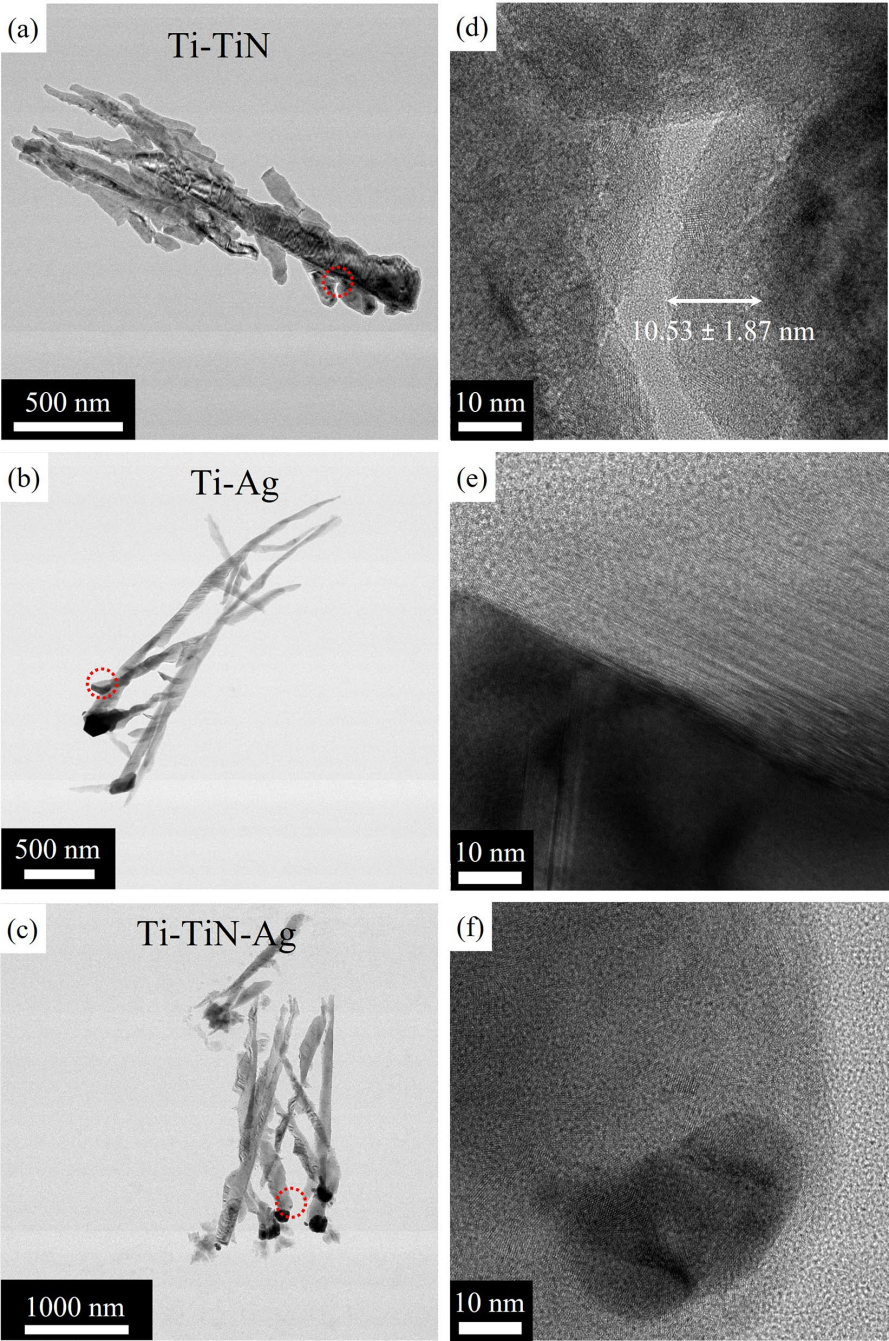
TEM image of branched Ti nanorod, (**a**) annealed for 4 h in N_2_, (**b**) capped with 100 nm of Ag on Ti, (**c**) capped with 100 nm of Ag on Ti annealed for 4 h in N_2_, and (**d**–**f**) is the magnified section of the circled spot of each nanorod.

In an effort to understand the relationship between the multiple nanorods and the branched Ti core, Fig. 2, shows a projection transmission electron microscope (TEM) images of a Ti–TiN, Ti–Ag, and Ti–TiN–Ag and their surface features. The combination of Fig. 2a,d, establishes that annealing Ti in the N_2_ atmosphere for 4 h produces a polycrystalline TiN layer 10.53 ± 1.87 nm averaged over 40 measurements around several samples. Our choice of TiN depth is based on the following two considerations. First, the depth needs to be close to the largest depth attainable by our system. Second, the depth of TiN needs to be sufficiently large to maximize the surface plasmon resonance properties while reducing the potential for oxidation at high temperatures. With these two considerations, we have tested a range of annealing times in N_2_: 1, 2, 4, 8, 16, and 36 h, and we found that the optimal depth correlates to the annealing time of 4 h. Beyond 4 h, we observe no significant changes in the depth which could be a result of a drop in annealing temperature from the sample stage in the presence of N_2_. Conversely, below 4 h, the depth of the TiN is reduced. Figure 2b,e and c,f shows the projection TEM of Ti–Ag and Ti–TiN–Ag nanorods. While the TiN layer is polycrystalline, the Ti core and Ag cap are single crystals.

**Figure 3:**
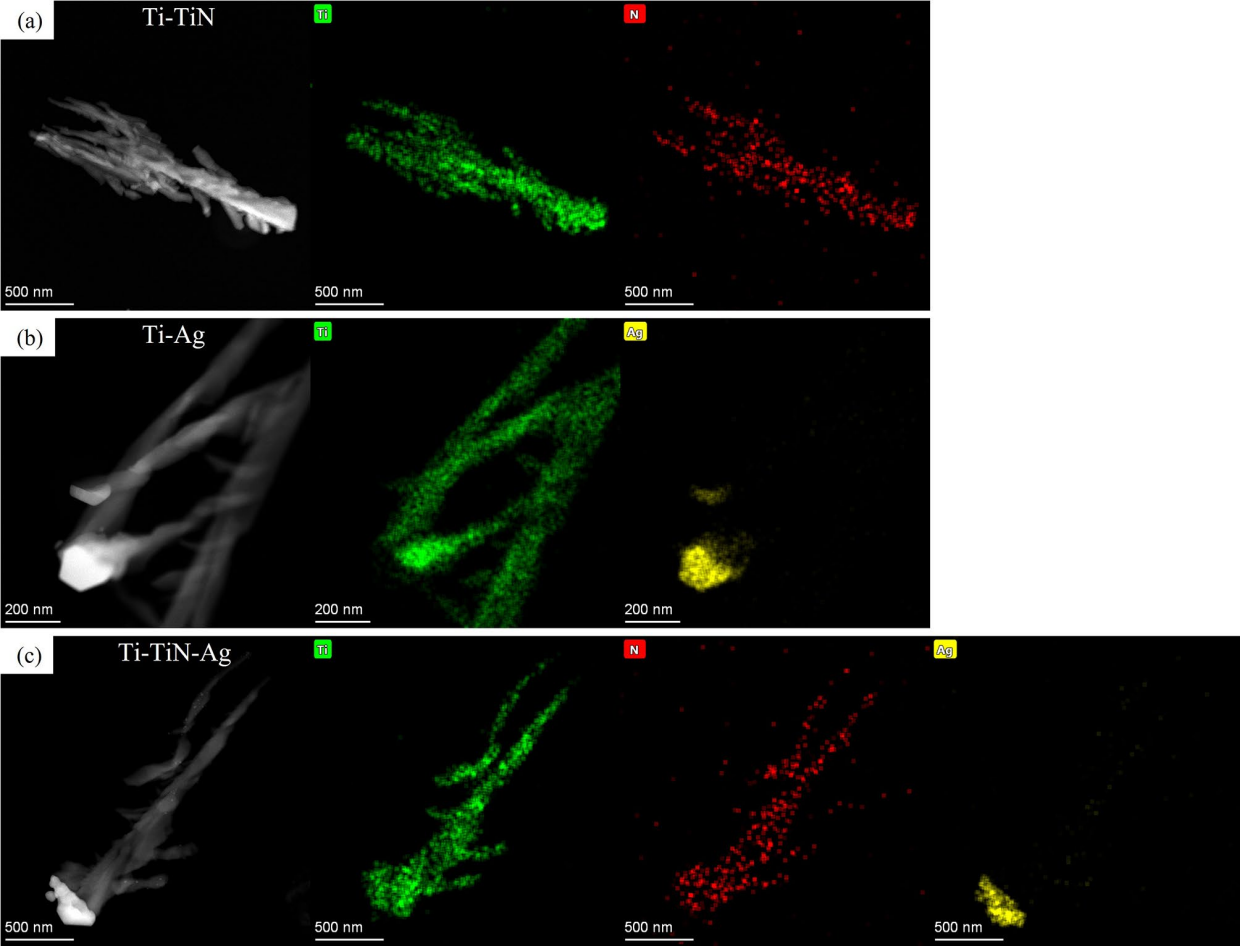
Map of element distribution in a cross-section of the branched Ti nanorods (**a**) annealed for 4 h in N_2_, (**b**) capped with 100 nm of Ag on Ti, (**c**) capped with 100 nm of Ag on Ti annealed for 4 h in N_2_.

Going beyond the morphology of the nanorods, we quantify the elemental composition of the branched nanorods. Figure 3, shows a high angle annular dark-field (HAADF) image of the Ti–TiN, Ti–Ag, and Ti–TiN–Ag samples and their corresponding energy dispersive X-ray (EDX) maps. The EDX maps present the distribution of chemical elements on the scanned surface of the nanorods and it unequivocally confirms the presence of Ti, N, and Ag respectively on the samples.

**Figure 4:**
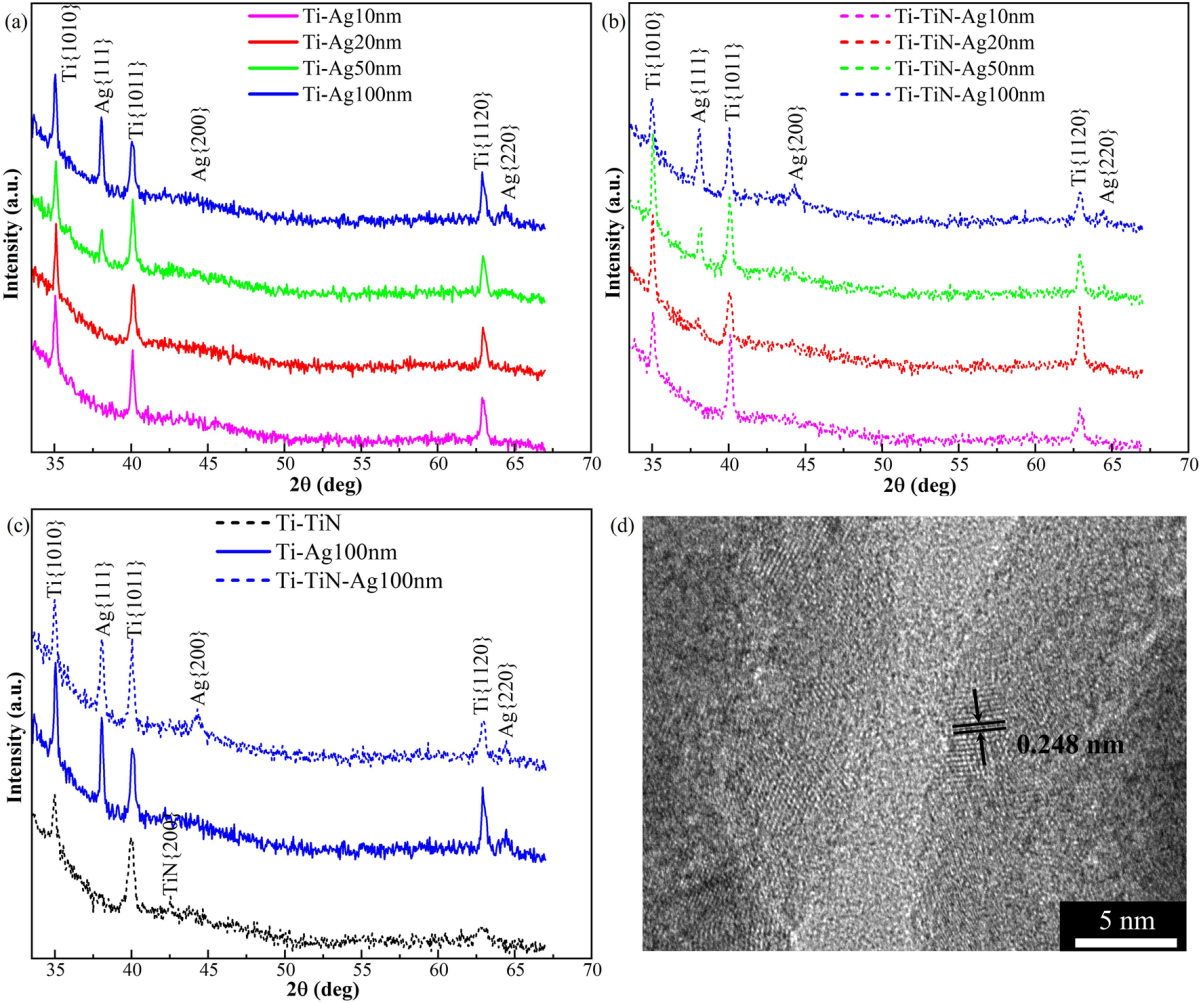
XRD intensity of nanorods (**a**) capped with Ag of 10, 20, 50, and 100 nm in thickness on Ti, (**b**) capped with Ag of 10, 20, 50, and 100 nm in thickness on Ti annealed for 4 h in N_2_, and (**c**) XRD intensity comparison as a function of angle 2θ for Ti–TiN, Ti–Ag and Ti–TiN–Ag nanorods of Fig. 1*.* (**d**) HRTEM image of a section of Fig. 2d showing the lattice d-spacing evidencing the presence of TiN.

The characterization of the crystal orientations of the nanorods using XRD is shown in Fig. 4. We identified the {$10\overline{1}0$}, {$10\overline{1}1$} and {$11\overline{2}0$} planes parallel to the substrate for the branched Ti core nanorod. That is, some nanorods have one of either plane parallel to the substrate surface. We also identify the presence of Ag {111}, {100}, and {110} planes with increasing intensity correlating to the increase in the Ag thickness. In passing, we also note that there is a detectable amount of TiN {100} in Fig. 4c, in agreement with the crystalline pattern observed in the HRTEM characterization in Fig. 4d where the lattice plane on a randomly selected crystal on the surface of the nanorod from Fig. 2d is 0.248 nm confirming a TiN {111} plane of fcc-TiN crystal (JCPDS card No. 38-1420)^26–31^.

**Figure 5:**
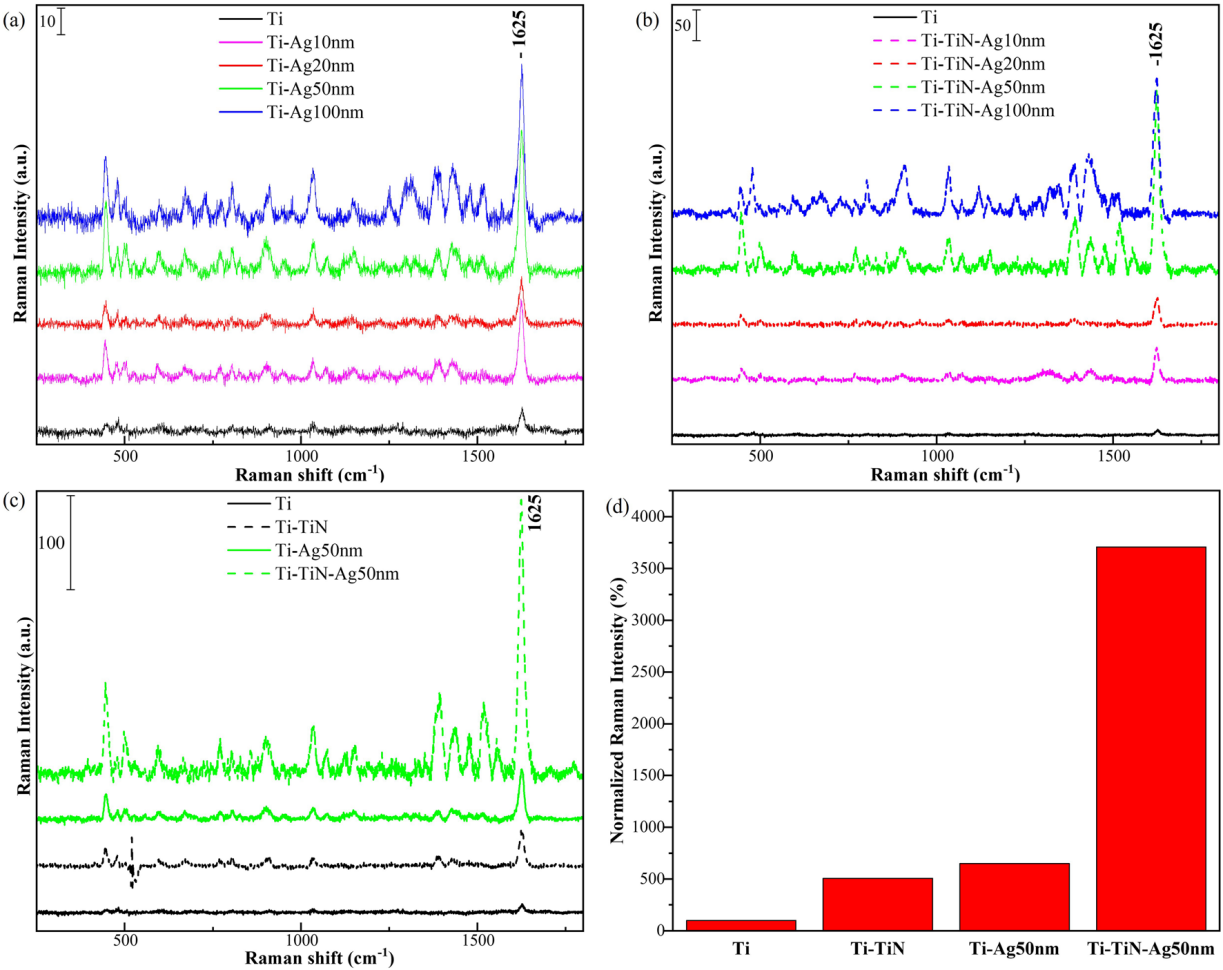
SERS spectra of 1.5 × 10^–6^ M MB molecules on Ti nanorods (**a**) capped with Ag of 0, 10, 20, 50 and 100 nm in thickness on Ti, (**b**) capped with Ag of 0, 10, 20, 50 and 100 nm in thickness on Ti annealed for 4 h in N_2_, (**c**) SERS spectra intensity comparison between as-deposited Ti, Ti–TiN, Ti–Ag 50 nm, and Ti–TiN–Ag 50 nm, and (**d**) normalized SERS peak intensities of MB molecules at 1625 cm^−1^ on as-deposited Ti, Ti–TiN, Ti–Ag 50 nm, and Ti–TiN–Ag 50 nm.

Based on the crystallographic variations of the branched nanorods, we expect that these substrates would exhibit different SERS sensitivity. Figure 5a shows the SERS spectra of 1.5 × 10^−6^ M MB molecules on the Ti–Ag nanorods with various thicknesses of Ag deposition in Fig. 1c. The spectrum on the as-deposited Ti nanorods substrate is included for comparison. It is clear that the SERS intensity increases with the increase in Ag capping thickness until it reaches 50 nm. A further increase to 100 nm of Ag capping thickness leads to a decline in the SERS intensity. This decline could be a result of a less strong EM field being generated between the gap regions of the top of the nanorods and their branches caused by the bridging observed in Fig. 1c. Figure 5b shows the SERS spectra of 1.5 × 10^−6^ M MB molecules on the Ti–TiN–Ag substrates of Fig. 1d. Similar to Ti–Ag substrates, the SERS sensitivity increases with the increase in Ag capping thickness until a maximum is reached at 50 nm. But it is clear that the SERS intensity is much higher with larger peak intensities than in Fig. 5a.

A comparison of the SERS sensitivity between the as-deposited Ti, Ti–TiN, Ti–Ag, and Ti–TiN–Ag is shown in Fig. 5c. Although Ti nanorods are not SERS sensitive and are prone to oxidation, the low Raman intensity detected could be a result of a charge transfer mechanism from TiO_2_^32–35^. The significantly high SERS enhancement from the Ti–TiN–Ag 50 nm is due to a variety of factors. Firstly, the TiN can excite surface plasmon resonance resulting in a strong EM field^19^. Second, due to the increased surface area of the branched titanium nanorods and the annealed film thickness of the TiN, the large surface area of Ti–TiN is conductive for adsorbing sufficient analyte molecules. Third, there exists a coupling effect of surface plasma from the combined charge transfer from Ag and TiN that provides a further electromagnetic and chemical enhancement for SERS as observed in nanoparticles of ZnO–TiN^36^, and Au–TiN^37^. Figure 5d shows the SERS peak intensities of the MB molecule at 1625 cm^−1^ which corresponds to the symmetric and asymmetric C–N stretching, as well as the C–C ring stretching, normalized by the peak intensity of the as-deposited Ti nanorod as a function of their coating compositions respectively on the nanorods. The normalized SERS peak intensity is calculated from the ratio of the peak intensity of the various SERS substrate and the peak intensity of the as-deposited Ti nanorods. It is encouraging that the annealing of the branched Ti nanorods for 4 h then capped with Ag increases the SERS sensitivity by 3708%, which represents a tremendous improvement over Ti–Ag—649% and Ti–TiN—507% increases.

## Conclusion

In this paper, we designed and fabricated various branched titanium nanorods using GLAD technique and successive nitridation technique as SERS substrate and analyzed them using SEM, TEM, XRD and by using a diluted solution of methylene blue and an excitation wavelength of 532 nm, we were able to analyze the optimal sensing performance. Based on these analyses, we make the following conclusions.

One, we have designed Ti–TiN–Ag nanorods that are oxidation-resistant and SERS sensitive. This design takes advantage of the large surface areas of Ti branched nanorods, oxidation resistance of TiN, and SERS sensitivity of TiN and Ag nanoparticles.

Two, the TiN layer is uniform between 10 and 11 nm. The deposition of Ag for nominally 50 nm results in the optimal SERS sensitivity, and bridging occurs as the deposition reaches 100 nm.

Three, the Ti branched nanorods and the Ag caps are single crystalline, while the TiN layer is polycrystalline around the nanorod.

## Methods

In this work, Ti nanorods with branches are deposited on Si{001} and microscope glass slide substrates by using an electron beam (e-beam) physical vapor deposition system under GLAD conditions. Details about the deposition method and conditions have been discussed in the previous report^6^. Before deposition, Si{001} and glass slide substrates are ultrasonically cleaned in a sequential bath of acetone, isopropyl alcohol, and deionized water for 30 min each and are then set to dry in atmospheric air. The cleaned Si{001} and glass slide substrates are attached to the stage set at a glancing angle of 87° with the direction of the incident flux and a temperature of 625 K. The nominal deposition rate is set to 0.5 nm/s. This rate is monitored with a quartz crystal microbalance (QCM) and it is achieved with a voltage of 10 kV and an emission current ranging from 70–120 mA. During deposition, the temperature of the substrate is increased by 3 K during deposition. The total nominal film thickness (with no porosity) is 1500 nm.

In the case of Ag capping, after the deposition of branched Ti nanorods, the source material target is switched to Ag in the deposition chamber without breaking the vacuum. The glancing angle is set to 87°, The substrate stage temperature is set to 625 K, and the deposition rate is decreased to 0.1 nm/s. The total nominal film thickness of Ag is 10, 20, 50, and 100 nm, respectively for each test. The branched Ti nanorods capped with Ag (represented as Ti–AgX—where X is the Ag cap nominal thickness) are set aside for characterization. To optimize the SERS sensitivity even further, another set of substrate samples was produced. Similar to Ti–AgX, after the deposition of branched Ti nanorods, N_2_ gas is introduced into the chamber for 15 min after shutting off both the turbomolecular pump and roughing pump. The branched Ti nanorod is annealed in N_2_ for 4 h with the substrate stage temperature still at 625 K. After 4 h, the roughing and turbomolecular pump are turned on so as to bring the chamber back to vacuum condition again and the source material target is switched to Ag in the deposition chamber. The glancing angle is kept at 87°, and the deposition rate is set to 0.1 nm/s. The total nominal film thickness of Ag is set as 10, 20, 50, and 100 nm, respectively for each test. The branched Ti nanorods annealed in N_2_ gas and capped with Ag (represented as Ti–TiN–AgX—where X is the Ag cap nominal thickness) are set aside for characterization.

The morphology and microstructural analysis of the prepared nanorods is performed using a high-resolution field scanning electron microscope (Hitachi S-4800, Tokyo, Japan). Under the accelerating voltage of 3 kV and with a working distance of 8 mm, the spatial resolution is 2 nm. The structure and elemental composition are characterized using a Cs-corrected transmission electron microscope (Thermo Fisher, TEM/STEM, FEI Titan Themis 300, Waltham, MA, USA). Under 300 kV, the spatial resolution reaches 0.07 nm and a diffraction detection diameter of 200 nm. Texture analysis is performed using X-ray diffraction (XRD, CuKa radiation of wavelength 0.154 nm, 40 kV, 44 mA, Rigaku ultima IV, Tokyo, Japan) for a sample size of 900 mm^2^ in area and 1.01 mm in total thickness of the Ti and the glass slide substrate. The nanorod dimensions are analyzed, measured, and processed using the ImageJ Processing Program^38,39^. SERS performance is characterized using a Raman Spectroscopy (Horiba Jobin Yvon HR800, Lille, France) at room temperature, with 1.5 × 10^−6^ M Methylene Blue (MB) as an analyte probing molecule. Raman spectra are collected based on an excitation laser of 532 nm, hole size of ~ 100 μm in diameter, acquisition parameters of 5 s exposure time, and reduced laser power of ~ 0.211 mW. The acquisition time and laser power were selected to avoid molecular degradation of the probing molecule induced by photochemical or thermal effects. Before SERS characterizations, all substrates are immersed into the MB solution for 30 min and then dried naturally in atmospheric air. The data collection time from each spectrum is set to be 5 s and each SERS spectrum is obtained by measuring and averaging the signals collected from three different spots on the same substrate.

## Supplementary Information

Supplementary Information.

## Data Availability

The generated and processed data for this study were provided as supplementary materials.
